# First Reported Chikungunya Fever Outbreak in the Republic of Congo, 2011

**DOI:** 10.1371/journal.pone.0115938

**Published:** 2014-12-26

**Authors:** Nanikaly Moyen, Simon-Djamel Thiberville, Boris Pastorino, Antoine Nougairede, Laurence Thirion, Jean-Vivien Mombouli, Yannick Dimi, Isabelle Leparc-Goffart, Maria Rosaria Capobianchi, Amelia Dzia Lepfoundzou, Xavier de Lamballerie

**Affiliations:** 1 Aix Marseille University, IRD French Institute of Research for Development, EHESP French School of Public Health, EPV UMR_D 190 "Emergence des Pathologies Virales" 13005, Marseille, France; 2 Centre National de Transfusion Sanguine, Brazzaville, Republic of Congo; 3 Laboratoire National de Santé Publique, Brazzaville, Republic of Congo; 4 French National Reference Centre for Arboviruses, IRBA Armed Forces Biomedical Research Institute, 13013, Marseille, France; 5 Laboratory of Virology, National Institute for Infectious Diseases "L. Spallanzani", Via Portuense 292, 00149, Rome, Italy; Agency for Science, Technology and Research - Singapore Immunology Network, Singapore

## Abstract

**Background:**

Chikungunya is an *Aedes* -borne disease characterised by febrile arthralgia and responsible for massive outbreaks. We present a prospective clinical cohort study and a retrospective serological study relating to a CHIK outbreak, in the Republic of Congo in 2011.

**Methodology and Findings:**

We analysed 317 suspected cases, of which 308 (97.2%) lived in the city of Brazzaville (66.6% in the South area). Amongst them, 37 (11.7%) were CHIKV+ve patients (*i.e.*, biologically confirmed by a real-time RT-PCR assay), of whom 36 (97.3%) had fever, 22 (66.7%) myalgia and 32 (86.5%) arthralgia. All tested negative for dengue. The distribution of incident cases within Brazzaville districts was compared with CHIKV seroprevalence before the outbreak (34.4% in 517 blood donors), providing evidence for previous circulation of CHIKV. We applied a CHIK clinical score to 126 patients recruited within the two first day of illness (including 28 CHIKV+ves (22.2%)) with sensitivity (78.6%) and specificity (72.4%) values comparing with those of the referent study in Reunion Island. The negative predictive value was high (92%), but the positive predictive value (45%) indicate poor potential contribution to medical practice to identify CHIKV+ve patients in low prevalence outbreaks. However, the score allowed a slightly more accurate follow-up of the evolution of the outbreak than the criterion "fever+arthralgia". The complete sequencing of a Congolase isolate (Brazza_MRS1) demonstrated belonging to the East/Central/South African lineage and was further used for producing a robust genome-scale CHIKV phylogenetic analysis.

**Conclusions/Significance:**

We describe the first Chikungunya outbreak declared in the Republic of Congo. The seroprevalence study conducted amongst blood donors before outbreak provided evidence for previous CHIKV circulation. We suggest that a more systematic survey of the entomological situation and of arbovirus circulation is necessary in Central Africa for better understanding the environmental, microbiological and sociological determinants of emergence.

## Introduction

Chikungunya virus (CHIKV, family *Togaviridae*, genus *Alphavirus*), is a classical arbovirus [1], discovered more than 50 years ago in East Africa [2]. It is responsible for acute febrile arthralgia -the name "Chikungunya" refers to the stooped posture characterising the infected patients [3]- with possible complications at the acute phase (*e.g.* neurological forms) but also possible long-term persistence of rheumatic symptoms [4], [5]. Over the last decade, CHIKV has been responsible for significant outbreaks involving millions of human cases in Eastern and Central Africa, the Indian Ocean, India and South-East Asia, and a limited - but alarming - number of autochthonous cases in Europe and, more recently, the Americas [6]. CHIKV is usually transmitted to humans by *Aedes* (Stegomyia) *aegypti* mosquitoes [7]. However, since 2005, it has been shown that an increasing number of transmission cases implicated *Aedes* (Stegomyia) *albopictus* mosquitoes. In a previous study [8], we provided three distinct examples (in the Indian Ocean islands, India and Central Africa) of the independent acquisition of a single adaptive mutation in CHIKV that, when exposed to *Aedes albopictus*, acquired a mutation in the envelope gene (E1, A226V) that has been reported to provide a selective advantage for the virus to be transmitted by this mosquito [9], [10].

CHIKVs are currently subdivided into three genotypes (Asian, East/Central/South African (ECSA), and West African) and the genome consists of a single positive strand of RNA that encodes four non-structural proteins involved in virus replication and pathogenesis, and five structural proteins that compose the virions.

Since its first isolation in 1952 in East-Africa [3], CHIKV has caused multiple outbreaks in the Old World. In both Africa and Asia, they were reported to be unpredictable, with a variable interval between each epidemic. In 2004, an outbreak occurred in Kenya, which spread over the Indian Ocean region with an unprecedented magnitude [11], [12]. Epidemics were subsequently reported in Senegal, Sudan, Cameroon, Gabon and for the first time in the Republic of Congo in 2011 [13].

The first ProMed-mail alert concerning a probable chikungunya outbreak affecting the Republic of Congo was published on June 13^th^ 2011 [14]. The first suspected cases appeared in early June in the district of Bacongo and Makélékélé in the south of Brazzaville. CHIKV infections were quickly confirmed by a laboratory in neighbouring Gabon [15]. According to the World Health Organisation (WHO), as of July 26^th^, a total of 11,083 suspected cases had been reported, of which 91% (10,066/11,083) originated from the Department of Brazzaville and 9% (1,117/11,083) from the Department of Pool (located in the south of the Republic of Congo). The most affected area in Brazzaville was the district of Makélékélé, which accounted for 65.7% of the total number of reported cases (7,279 cases) [16], [17].

Since 2006, a large amount of information regarding the clinical manifestations of CHIKV has been collected which relates to both "standard" and complicated presentations. Nevertheless, with a few exceptions (*i.e.*, the original CHIKV description, the Cameroon outbreak in 2006 and the Gabon outbreak in 2010), most clinical studies were conducted outside Africa [3], [18], [19].

Here, we present findings relating to the CHIK outbreak in the Republic of Congo in 2011. The study included three complementary objectives: *(i)* a clinical study in which we assessed a recently published clinical score, originally established in a different setting [20]; *(ii)* an epidemiological study based on the analysis of incident laboratory confirmed cases and a seroprevalence analysis conducted amongst blood donors in the pre-epidemic period; *(iii)* a molecular study, in which we produced complete sequences and a robust genome-scale phylogenetic analysis of the Congolese virus together with a recent dataset of complete sequences.

## Materials and Methods

### Design

We present a CHIKV retrospective, cross-sectional, serological study followed by a prospective clinical cohort study performed during the CHIK outbreak in the Republic of Congo in 2011.

#### Setting

This study took place in Brazzaville, the political and administrative capital city of the Republic of Congo. Brazzaville is located in the south of the country, on the north shore of the Congo River across from Kinshasa (Democratic Republic of Congo). Brazzaville was, at the onset of the study, divided into seven districts (namely: Makélékélé, Bacongo, Poto-Poto, Moungali, Ouenze, Talangai and Mfilou). To facilitate our analysis, we grouped the districts into three areas according to the geographical location (Makélékélé, Bacongo - South), (Poto-Poto, Moungali, Ouenze, Mfilou - Center) and (Talangai - North).

According to the "Centre National de la Statistique et des Etudes Economiques" (CNSEE), on 1^st^ January 2011, 3,697,490 people lived in the Republic of Congo of which 1,373,382 lived in Brazzaville. The characteristics and spatial distribution of the general population of Brazzaville in the different districts is presented in the table 1.

**Table 1:** Characteristics of the populations studied in Brazzaville with reference to the general population.

	Serological study (N = 517)	Clinical study (N = 308)	General population (N = 1 373 382)
**Age**			
Mean	33.85	29.17	21.00
Median	32	29.00	23.44
Extremes	18–60	1–68	0–90+
SD	10.58	15.20	16.99
**Age group; n (%)**			
≤17	-	69 (22.4)	578 156 (42.2)
18–29	221 (42.7)	86 (27.9)	351 764 (25.6)
30–39	140 (27.1)	72 (23.4)	209 414 (15.2)
40–49	101 (19.5)	47 (15.3)	120 971 (8.8)
50–59	55 (10.6)	34 (11.0)	63 119 (4.6)
60–69	-	-	30 805 (2.2)
≥70	-	-	19 153 (1.4)
**Gender**			
M/F	391/126	133/173	677 599/695 783
Gender ratio	3.1	0.76	0.97
**Occupation**			
Work/Employed	293 (56.7)	77 (25.0)	
Student	100 (19.3)	118 (38.3)	
Unemployed	124 (24.0)	87 (28.2)	
Children (<13yo) not attending school	-	26 (8.4)	
**Districts of Brazzaville; n (%)**			
Makelekele	90 (17.4)	175 (56.8)	298 292 (21.7)
Bacongo	124 (24.0)	30 (9.7)	98 782 (7.2)
Poto-Poto	30 (5.8)	6 (1.9)	93 106 (6.8)
Moungali	35 (6.8)	10 (3.2)	166 719 (12.1)
Ouenze	54 (10.4)	9 (2.9)	182 057 (13.3)
Talangai	130 (25.1)	58 (18.8)	337 986 (24.6)
Mfilou	54 (10.4)	20 (6.5)	196 440 (14.3)
**Brazzaville by zone; n (%)**			
South	214 (41.4)	205 (66.6)	397 074 (28.9)
Centre	173 (33.5)	45 (14.6)	638 322 (46.5)
North	130 (25.1)	58 (18.8)	337 986 (24.6)

In Brazzaville, healthcare facilities consist of six public hospitals and numerous integrated care centres.

### Population and procedures

#### Seroprevalence study

To estimate the seroprevalence of CHIKV infection in the population of Brazzaville before the 2011 CHIKV outbreak, sera from blood donors collected in Brazzaville before June 2011 (from March 29th to May 26th) were provided by the national Congolese blood bank (Centre National de Transfusion Sanguine, CNTS).

#### Clinical study

The CNTS and the national laboratory of public health (Laboratoire National de Santé Publique, LNSP) of the Republic of Congo designed and conducted this clinical study in collaboration with the French institute for research and development (IRD).

The study started on June 2nd 2011 [week (W) 22:W22] and finished on July 28th 2011 (W30). During this period, all patients suspected of CHIK who consulted any of the health care facilities in Brazzaville were eligible for enrolment.

A suspected case was defined as a patient with at least one of the following symptoms: fever, arthralgia, myalgia, headache or rash. All suspected cases that reported such symptom(s) in the previous eight days and agreed to participate to the study were included. Were excluded patients who did not report any symptom of the case definition and those who had symptom(s) but did not will to participate.

After inclusion, medical examination and interview were completed, data were collected on a standardised questionnaire [20], which included age, gender, residence, time of onset, concomitant treatments, intensity of symptoms and location of arthralgia. The quality of sleep was assessed by self-reported visual analogic scale (VAS) from “very bad” (VAS = 0) to “very good” (VAS = 100).

Venous blood samples were drawn using two 5 mL EDTA and one 4 mL plain tubes which were immediately centrifuged. Serum/plasma aliquots were kept at −80°C.

Virological investigations were retrospectively performed in the UMR_D 190 laboratory, in Marseille, France.

The case definition of CHIKV positive patients (CHIKV+ves) relied on retrospective laboratory confirmation of CHIKV infection (positive CHIKV specific RT-PCR).

The patients who were included within the two first days of illness were used to compare the clinical presentation of CHIKV+ve and CHIKV negative (CHIKV-ve) patients.

### Ethical considerations

Oral (patients in Emergency units who accepted to fill the study questionnaire) and/or written (blood donors) consent was obtained for each person enrolled in the study. The project and the consent procedure were approved by the Congolese Research in Health Sciences Ethics Committee as an extension of an ongoing study of the arbovirus epidemiology in Republic of Congo (N° 00000065 DGRST/CERSSA).

Negative control serum samples for serological analyses (PRIAM study) were collected after obtaining written informed consent from all participants.

### Laboratory analysis

#### Molecular diagnosis

Nucleic acids were extracted from serum samples using the EZ1 Virus Mini Kit v2 and an EZ1 advanced XL Biorobot workstation (Qiagen). Detection of viral genomes was performed using a one-step TaqMan real time RT-PCR procedure and the kit SuperScript III Platinum One-Step Quantitative RT-PCR System with ROX (Life Technologies) for the CHIKV and the dengue virus (DENV), as previously described [21], [22], [23]. In addition, plasma samples were tested for the presence of *Plasmodium falciparum* genomes using a previously reported real time PCR procedure targeting the *P. falciparum* aquaglyceroporin gene [21], [22], [23] and the Platinum Quantitative PCR SuperMix-UDG (Life Technologies).

#### Serological analysis

Specific IgG against CHIKV were detected using an enzyme-linked immunosorbent assay (ELISA) method and a standard operating procedure [24]. The National Reference Centre for arboviruses provided a panel of 328 serum samples from blood donors living in metropolitan France, which tested negative for IgG to CHIKV (PRIAM study). This panel was used to determine the cut-off of the test (established as the mean of the adjusted OD values of these negative controls plus two standard deviations).

#### Virus isolation

Propagation onto cell cultures was carried out in a biosafety level 3 laboratory. Virus isolation was attempted using serum samples with a positive RT-PCR result by inoculating 100 µL of serum directly onto C6/36 *Aedes albopictus* cells. Viral growth was identified by combining the observation of gross cytopathic effect and a specific molecular detection using the same real time specific RT-PCR as described above.

#### CHIKV genome sequencing

A complete genome sequence (excluding the first 18 nucleotides of the 5'UTR and the 20 nucleotides upstream the polyA tail) was produced from the first passage of a CHIKV strain isolated from a 3-year old boy presenting with fever and dermatological signs and who was living in the district of Makélékélé, Brazzaville. Nucleic acids were extracted from cell culture supernatants using the EZ1 Virus Mini Kit v2 and the EZ1 Biorobot (Qiagen). Primers previously designed to sequence the LR2006 OPY1 CHIKV strain were used to generate PCR products with the Access RT-PCR System (Promega). Amplicons were purified and sequenced using the Sanger method and the BigDye Terminator v3.1 Cycle Sequencing Kit on an ABI Prism 31310X Genetic Analyser sequencer (both from Life technologies). Analysis of sequencing chromatograms was performed using the Sequencher 4.9 software (Gene Codes Corporation).

### Statistical analysis

To describe the clinical characteristics of the population studied, we performed a bivariate analysis and p-values were determined for qualitative variables with a Fisher's exact test; for continuous variables a Mann Whitney nonparametric test was used. Correlations were assessed using the Spearman nonparametric test (rho coefficient, r). The patients included within the 2 first days of illness were used to compare CHIKV+ve patients to CHIKV-ve patients.

With regard to a previous study [20], we evaluated a clinical score for the diagnosis of CHIK amongst patients included within the two first day of illness. This score was previously presented from a cohort of suspected CHIK outpatients during the Reunion outbreak in 2006. Our score was based exclusively on clinical data and particularly on the presence or absence of 3 symptoms: arthralgia in at least one hand, arthralgia in at least one wrist and minor or absent myalgia [20]. In the Reunion Island referent population, the clinical score had a Sensitivity of 76%, a Specificity of 73%, a PPV and NPV of 87% and 55% respectively. The probability (p) of having been infected by CHIK was estimated, using logistic regression, as follows:

p = 1/(1+exp(1.609)6exp(21.4506MCP+)6exp(21.7326W+)6exp(22.0446MYOPAIN)) where each covariate was validated if equal to 1, otherwise 0.

Sensitivity (Se), specificity (Sp), predictive values –positive (PPV) and negative (NPV) – were calculated and yielded a Receiver Operating Characteristic (ROC) curve and the area under the curve (AUC).

All statistical analyses were performed with the IBM SPSS statistic 21 software. The alpha level of significance chosen was 0.05.

### Phylogenetic analysis

The two open reading frame (ORFs) of available complete CHIKV genomes were manually extracted, concatenated using Mega 5.1 [25] and aligned with ClustalW [26] according to the amino acid sequence. This included sequences available from the GenBank database, together with the sequence of Congolese isolate, also a number of previously unreleased sequences from the Indian Ocean Region and Italy. Two codon positions were removed from the alignment: *(i)* residue 1,857 of the non-structural polyprotein (associated with read through within the NSP4 protein) which is non-informative for phylogenetic reconstructions and *(ii)* residue 1,035 of the structural polyprotein (associated with the single mutation providing an assumed selective advantage for transmission by *Aedes albopictus* at E1 position 226) since variability at this site may be driven by convergent adaptive evolution [8].

The phylogenetic tree was inferred using the maximum likelihood method with the RAxML 7.3.0 program [27]. Mega 5.1 was used to select the best-fit model for the analysis (GTR model of nucleotide substitution with gamma-plus-invariant-sites-distributed rates of change among sites) [25] and a bootstrap analysis was performed (1,000 replicates). The tree was edited with FigTree v1.3.1.

## Results

### Serological and Clinical studies

#### Population studied

Overall, 517 serum samples of blood donors were collected and analysed before the outbreak, between March 29th, 2011 and May 26th, 2011. The gender ratio (M/F) was 3.1 and median age of 32 (Table 1).

From June 2nd 2011 (W22) to July 28th 2011 (W30), 317 suspected cases of CHIKV were recorded, of which 308 (97.2%) lived in the city of Brazzaville and, for the majority of cases, in the district of Makélékélé (175/308, 56.8%) (Table 1). Nine patients originated from other departments: eight (2.5%) from the Pool department and one (0.3%) from the north of country. The population studied was young (median age: 30) and mostly urban. The gender ratio (M/F) was 0.77 and 135 (42.6%) patients were enrolled within 2 days from the onset of the disease.

Three distinct periods corresponding the number of inclusions of suspected cases were defined: 45 (14.2%) patients during W22–26, 132 (41.6%) during W27–28 and 140 (44.2%) during W29–30.

**Figure 1:**
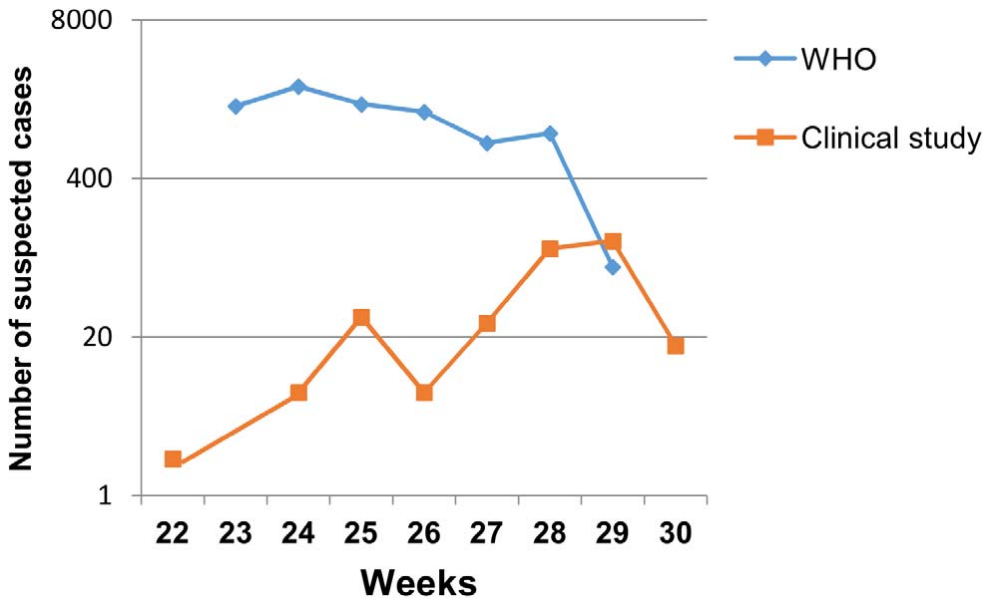
Weekly distribution of CHIK suspected cases in Republic of Congo, 2011. Note: The reuse figures of WHO curve were initially published under an open-access license (CC-BY)

The inclusion curve of suspected cases of our study was compared with the WHO epidemic curve (Fig. 1). 135 patients were included within the two first days of illness, 100 between days 3 and 4, and 82 between days 5 and 7.

#### Geographical distribution of cases

The distribution of cases within the seven districts of Brazzaville was studied and compared with the prevalence of antibodies to CHIKV in blood donors before the outbreak.

**Figure 2:**
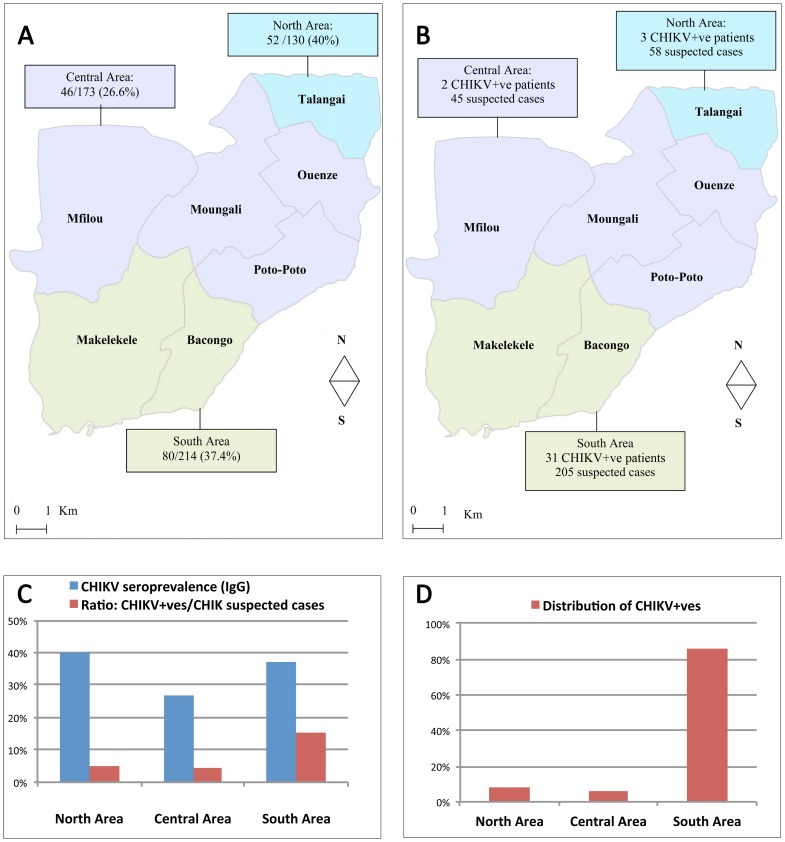
In three areas of Brazzaville city: distribution of CHIKV seropositive (IgG) blood donors before the outbreak (A); distribution of suspected and confirmed (CHIKV+ve patients) cases during the outbreak (B); summary of seroprevalence and incidence data (C); distribution of CHIKV+ve cases (D). Of note, suspected cases included patient who reported evocative symptoms in the previous eight days. Since the duration of viraemia is usually shorter, this may lead to underestimate the ratio CHIKV+ve/CHIK suspected cases, but does not hamper the comparison of values amongst the three areas considered.

Among the 517 blood donors collected, 178 (34.4%) showed positive IgG against CHIKV. The seropositivity rate was 16.3% (29) in Makélékélé, 28.7% (51) in Bacongo, 6.2% (11) in Poto-Poto, 2.8% (5) in Moungali, 7.3% (13) in Ouenze, 9.6% (17) in Mfilou, and 29.2% (52) in Talangai. Districts were grouped into three areas. The seropositivity rate was, in decreasing order: 40% (52/130) in the North Area (Talangai district); 37.4% (80/214) in the South Area (Makélékélé and Bacongo districts); 26.6% (46/173) in the Central Area (Poto-Poto, Ouenze and Mfilou districts) (Fig. 2A). The distribution of suspected and incident cases of CHIKV is presented in Fig. 2B and reveals that a vast majority of cases occurred in the South district. For each area, the CHIKV seroprevalence before the outbreak, the percentage of suspected cases that were confirmed and the contribution to the total number of positive cases are presented in Fig. 2.

#### Molecular diagnosis

Amongst the 317 patients studied, 37 (11.7%) had a positive PCR result for CHIKV (CHIKV+ve patients). Of the 135 patients who enrolled early (two first days), 32 were CHIKV+ve (23.7%). All serum samples were negative for DENV genome detection and five CHIKV-ve samples had positive qPCR detection for *P. falciparum*.

**Figure 3:**
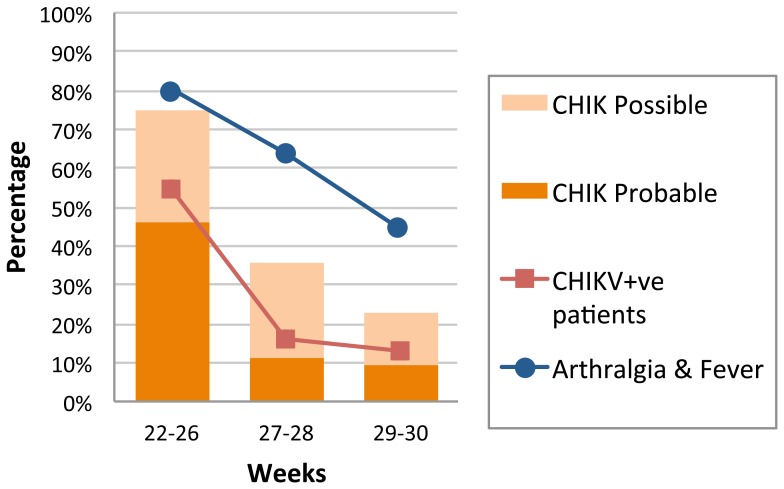
Weekly trend of CHIKV+ve patients (confirmed cases), "CHIK probable" and "CHIK possible" cases (according to clinical diagnostic score) and "arthralgia + fever" cases, Republic of Congo, 2011. Curves for “CHIKV+ve patients” and "arthralgia + fever" were created with the 135 patients enrolled within the 2 first days of the illness. Nine patients were excluded for the calculation of the clinical score (incomplete information regarding myalgia or arthralgia).

The evolution of the positivity rate (*i.e.*, during W22–26, W27–28 and W29–30) is shown in Fig. 3. It drops from ∼55% during the first 5 weeks of the outbreak to less than 15% during the last two weeks studied.

#### Clinical description of CHIKV+ve patients

Amongst the 37 CHIKV+ve patients, the median age was 33 years old; the gender ratio (M/F) was 0.95. Detection of CHIK occurred in patients consulting on average 1.8 days (range, 0 to 5 days) after the onset of symptoms; 32 (86.5%) consulted from day 0 to day 2, four (10.8%) on day 3, and one (2.7%) on day 5.

At the time of the consultation, 36 (97.3%) patients had fever, 22 patients (66.7%) declared myalgia and 32 (86.5%) arthralgia, including 18 (48.6%) with an intensity assessed as important. Amongst the 32 patients with musculoskeletal pain, joint pain was mostly polyarticular: on average, 5 joints per patient were affected (SD 3) and 18 (56.2%) had more than three joints that were affected. Arthralgia occurred preferentially in the small upper joints (wrists and hands) followed by knees, ankles and feet.

Five (13.5%) patients declared no arthralgia but had fever and dermatologic or digestive disorders.

Skin lesions were noticed in 16 (43.2%) patients, 14 (37.8%) of which also had pruritus. Digestive symptoms, consisting of diarrhoea, nausea and vomiting were described in a limited number (7) of cases (18.9%).

General symptoms were also recorded: headache in 23 (63.9%) cases, asthenia in 35 (94.6%), and chills in 12 (32.4%). The quality of sleep was severely impacted with 27 (73%) patients reporting "very bad".

In bivariate analysis, an increase of age was correlated with a higher intensity of headache (p<0.001, r = 0.687), myalgia (p<0.01, r = 0.523), poor quality of sleep (p<0.05, r = 0.345), and a lower frequency of diarrhoea (p<0.001, r = −0.575). Gender was not associated with specific symptoms or severity of symptoms.

#### Clinical description of patients with positive *P. falciparum* detection

Amongst the five patients with positive sample for *P. falciparum*, two (40%) complained about cervical or lumbar arthralgia. None reported arthralgic pain for small joints (*i.e.*, wrists or hands.).

#### Comparison between CHIKV+ve and CHIKV-ve patients at the early stage of disease

A vast majority of CHIKV+ve patients (86.5%) consulted before day 3. Accordingly, a comparison between CHIKV+ve and CHIKV-ve patients was performed for those who consulted within 2 days from the onset of symptoms (*i.e.*, 32 CHIKV+ve and 103 CHIKV-ve patients). The main findings are summarised in Table 2.

**Table 2:** Demographic and clinical characteristics of CHIKV+ve and CHIKV-ve patients included at Day 1-Day 2.

		CHIKV+ve (n = 32)	CHIKV-ve (n = 103)	p-value	Odd Ratio (CI 95%)
**Age**; mean (SD)		28.88 (18.13)	27.04 (16.91)		
**Sex; n (%)**	M	15 (46.9)	35 (34.0)	0.212	0.58 (0.26;1.31)
	F	17 (53.1)	68 (66.0)		
**Fever; n (%)**		31 (96.9)	100 (97.1)	1.0	0.93 (0.093;9.26)
**Arthralgia; n (%)**		27 (84.4)	56 (54.4)	0.0031	4.53 (1.62;12.69)
Number mean (SD)		3.81 (2.96)	1.83 (2.32)		
Intensity; n (%)				0.000001	ND
	Absent	5 (15.6)	47 (45.6)		
	Minor	1 (3.1)	11 (10.7)		
	Moderate	10 (31.3)	38 (36.9)		
	Important	16 (50.0)	7 (6.8)		
Localisation; n (%)	Wrist	25 (78.1)	36 (35.0)	0.00003	6.65 (2.62;16.86)
	Hands (MCP+PIP)	24 (75.0)	29 (28.2)	0.000003	7.66 (3.09;18.98)
	Ankles	14 (43.8)	22 (21.4)	0.021	2.86 (1.23;6.65)
	Feet	12 (37.5)	10 (9.7)	0.00059	5.58 (2.12;14.7)
	Knees	12 (37.5)	25 (24.3)	0.174	1.87 (0.80;4.36)
	Cervicalgia	10 (31.3)	22 (21.4)	0.341	1.67 (0.69;4.05)
	Lombalgia	10 (31.3)	20 (19.4)	0.222	1.88 (0.77;4.61)
	Shoulders	8 (25.0)	12 (11.7)	0.086	2.53 (0.93;6.88)
	Elbows	7 (21.9)	12 (11.7)	0.155	2.12 (0.76;5.96)
**Headache; n (%)**		21 (67.7)	58 (56.9)	0.305	1.59 (0.68;3.72)
**Asthenia; n (%)**		32 (100.0)	93 (90.3)	0.117	ND
**Myalgia; n (%)**		18 (64.3)	56 (57.1)	0.524	1.35 (0.56;3.22)
Moderate or Important		15 (53.6)	36 (36.7)	0.129	1.99 (0.85;4.64)
**Shiver; n (%)**		11 (34.4)	58 (56.3)	0.042	0.41 (0.18;0.93)
**Dermatological signs; n (%)**		14 (43.8)	33 (32.0)	0.288	1.65 (0.73;3.72)
**Nausea or Vomiting; n (%)**		7 (21.9)	23 (22.3)	1.00	0.97 (0.37;2.54)
**Diarrhoea; n (%)**		7 (21.9)	19 (18.4)	0.798	1.24 (0.47;3.28)
**Respiratory signs; n (%)**		1 (3.1)	4 (3.9)	1.00	0.80 (0.09;7.41)
**Haemorrhagic signs; n (%)**		0 (0.0)	1 (1.0)	1.00	
**Pruritus; n (%)**		11 (34.4)	16 (15.5)	0.025	2.85 (1.15;7.03)
**Burn under the feet; n (%)**		4 (12.5)	2 (1.9)	0.028	7.21 (1.26;41.44)
**Quality of Life; n (%)**					
Capacity to perform normal activity				0.129	ND
	Very Bad	9 (28.1)	16 (15.5)		
	Medium	23 (71.9)	86 (83.5)		
	Very good	0 (0.0)	1 (1.0)		
Health Status				0.676	ND
	Very Bad	2 (6.3)	5 (4.9)		
	Medium	30 (93.8)	97 (94.2)		
	Very good	0 (0.0)	1 (1.0)		
Quality of Sleep				0.009	ND
	Very Bad	25 (78.1)	53 (51.5)		
	Medium	7 (21.9)	49 (47.6)		
	Very good	0 (0.0)	1 (1.0)		
**Use of Paracetamol; n (%)**		32 (100.0)	102 (99.0)	1.00	ND
**Use of NSAIDs; n (%)**		11 (34.4)	19 (18.4)	0.086	2.32 (0.96;5.60)

No significant difference in age or gender ratio was identified. CHIKV+ve patients complained more frequently about arthralgia, notably for small joints such as wrists, hands, ankles, feet than CHIK-ve patients. The number and intensity of joint pains were also higher in CHIKV+ve patients. CHIKV+ve patients notified more frequently pruritus or "burn under the feet" and an impaired quality of sleep compared to CHIKV-ve patients. No significant difference between patients who had myalgia was noticed. By contrast, CHIKV+ve patient less frequently reported shiver and no significant difference was observed for dermatological and gastro-intestinal symptoms.

#### Diagnostic score

A total of 135 patients was enrolled within 2 days from the onset of the disease. Nine patients were excluded (incomplete informations regarding myalgia or arthralgia) and the clinical score was therefore applied to 126 patients: 23 (18.3%) were "probable" cases, 26 (20.6%) "possible" cases, and 77 (61.1%) "not probable" cases. Prevalence was (28/126) 22.2% in this population. The performance of the score for detecting "possible + probable" cases was as follows: sensitivity, 78.6%; specificity, 72.4%; PPV, 45%; NPV, 92.2%. The ROC curve had an AUC = 0.76 (p<0.001). In comparison, amongst the 135 patients enrolled within the 2 first days of the illness, the sensitivity, specificity, PPV and NPV of the criterion "arthralgia and fever" in this population were as follows: 85.7%, 44.9%, 30.8%, 91.7% respectively.

The percentages of "possible" and "probable" cases, in patients reporting "arthralgia and fever", and of CHIKV+ve patients (laboratory confirmed) was followed (W22–26, W27–28, W29–30) and are presented in Fig. 3. The distribution of "probable" and "possible + probable" cases faithfully follows that of confirmed cases (with a slight underestimation and overestimation of the incidence rate, respectively), providing a more acute estimate of the actual epidemiological evolution than the simple "fever + arthralgia" criterion.

### Phylogenetic analysis

**Figure 4:**
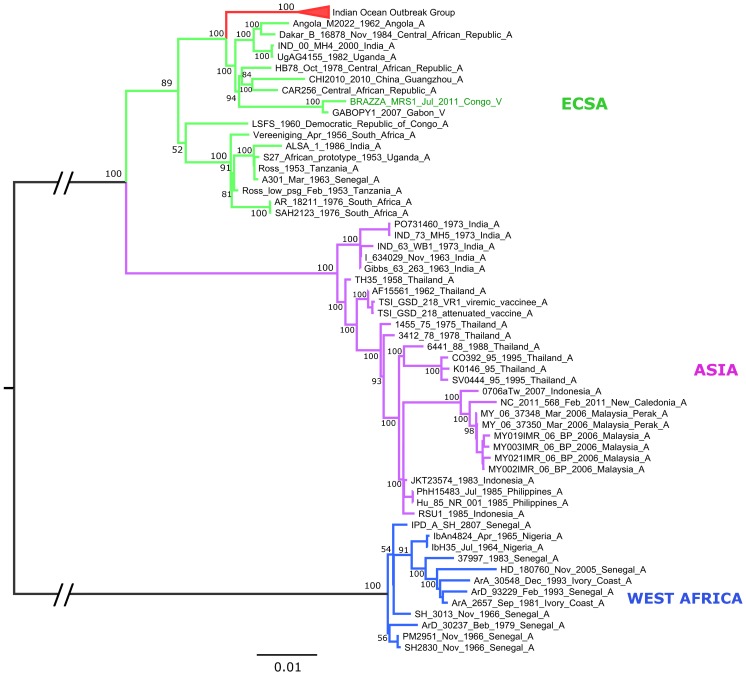
Maximum likelihood phylogenetic reconstruction using CHIKV nucleotide complete coding sequences.

Phylogenetic analysis of the complete sequence of isolate Brazza_MRS1 (Genbank accession number pending: KP003813) revealed that it belongs to the East/Central/South African (ECSA) lineage (Fig. 4). As previously reported, this lineage splits into three distinct clades [28]: the first (ECSA1) contains Ross and S27-African prototype strains and a variety of strains from West, Central, East and South Africa; the second (ECSA2) contains a majority of strains originating from Central Africa (*e.g.*, HB78 and UgAG4155 strains); the third contains strains which recently emerged in the Indian Ocean basin and in Asia (called Indian Ocean Outbreak Group in Fig. 4). Strain Brazza_MRS1 falls into the ECSA2 group and is closely related to GABOPY1 (KP003812), a strain isolated in 2007 during an outbreak in Gabon. This grouping is supported by 100% bootstrap values for the clade itself and for the sister relationship between Brazza_MRS1 and GABOPY1. The concatenated sequences of the two ORFs of these two strains are 99.6% (11,120/11,166 nt) and 99.5% (3,703/3,722 aa) identical at the nucleotide and amino acid levels, respectively. The E1-A226V mutation, associated with adaptation to the mosquito *Aedes albopictus*, is present in both strains [9], [10].

**Figure 5:**
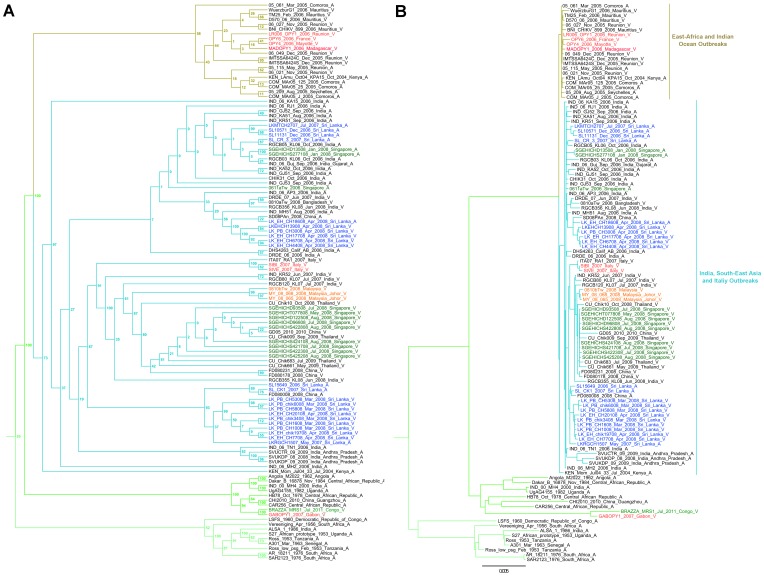
Detailed phylogeny of CHIKV genotype ECSA. Subtrees include all nucleotide sequences of CHIKV genotype ECSA extracted from the maximum likelihood tree presented in the manuscript (**Fig. 4**). **Panel A** includes a cladogram with boostrap values (only topology is displayed); **a** phylogenetic tree in which horizontal lines are proportional to genetic distances is presented in **panel B**. Sequence information for each virus corresponds to virus strain name/date of isolation (when available)/country of isolation/symbol for the amino-acid at the position E1-226. The sequence from the CHIKV strain isolated and completely sequenced in the present study was named "BRAZZA_MRS1_Jul_2011_Congo_V". The sequences in red were also sequenced by the UMR_190_EPV (Marseille, France).

The complete subtree with all CHIKV genotype ECSA sequences is available in Fig. 5. Regarding strains associated of the Indian Ocean Outbreak Group, the tree topology is similar to that proposed by Volk and collaborators (2010) and supports the earlier hypothesis [8] that the Indian Ocean and Indian subcontinent outbreaks emerged independently, presumably from Kenyan or early Comoran *Aedes aegypti*-associated CHIKV variants.

We established additional full coding sequences of isolates from Mayotte (OPY4, 2006, KP003809) and Madagascar (MADOPY1, 2006, KP003808) and included them in genome-scale analysis. Like all other isolates characterised to date [9], the Mayotte strain includes the A226V mutation, in contrast with two 2005 available sequences from other Comoran islands. Since both *Aedes albopictus* and *Aedes aegypti* mosquitoes are present in Mayotte, this may imply either that original A226 strains from Comoros became rapidly adapted to *Aedes albopictus*, or alternatively, that the Mayotte CHIKV strains originated from Reunion island or Mauritius where a rapid shift to *Aedes albopictus*-associated strains was observed [9].

The MADOPY1 strain was isolated in 2006 from a French patient returning from Madagascar. Interestingly, this strain includes the A226V mutation. This is in contrast with eight 2006 Malagasy isolates characterised by Schuffenecker and collaborators [9]. Since both *Aedes albopictus* and *Aedes aegypti* mosquitoes are present in distinct areas of Madagascar, this finding may indicate that both *Aedes albopictus-* and *Aedes aegypti*-associated strains have co-circulated in Madagascar as early as 2006. The presence of *Aedes albopictus*-associated virus strains has been subsequently confirmed in 2007 in a traveller returning from Madagascar [29].

In addition, we analysed and included isolates made from autochthonous transmission of CHIKV in Europe. Strain OPY6 (2006) was isolated from a case of autochthonous nosocomial infection in a nurse in metropolitan France [30]. The index case was a patient returning from Reunion Island. As expected, OPY6 (KP003807) sequence includes the A226V mutation and is grouped with the other 2006 Indian Ocean CHIKV sequences. Other cases were identified in Italy during the 2007 outbreak. Strains StVE (KP003810) and StBI (KP003811) (isolated from human cases of autochthonous CHIKV transmission that occurred in Northern Italy) form a cluster with the previously characterised ITA07-RA1 strain (ML bootstrap: 100%) (Fallacara and Bonilauri 2008, unpublished), most likely indicating a single viral introduction event and subsequent spread in Italy in 2007. All of them contain the A226V mutation, in accordance with the potential local exclusive transmission by *Aedes Albopictus* [31].

## Discussion

The 2011 chikungunya outbreak investigated in the current study was the first declared in the Republic of Congo. However, numerous epidemics have been reported in neighbouring countries during the last 50 years (Angola in 1962, 1970, 1971; Central African Republic in 1978, 1984; Democratic Republic of Congo in 1960, 1999–2000; Cameroon in 2006 and Gabon in 2007–2012) [5]. There is therefore strong evidence for sustained CHIKV circulation in this African region. This is confirmed by the seroprevalence study that we conducted in blood donors three months before the onset of the outbreak and which revealed a 34.4% global prevalence of antibodies to CHIKV in Brazzaville city.

Little information is available regarding the decisive factors, environmental characteristics and epidemiological dynamics of chikungunya circulation in the Republic of Congo. The molecular analysis of a chikungunya strain isolated during the 2011 outbreak suggested, based on the presence of the A226V adaptive mutation, that the virus could be transmitted by *Aedes albopictus*, in agreement with previous findings in Gabon [32]. An entomological study conducted in the Makélékélé and Mfilou districts during the CHIK outbreak identified CHIKV in both *Aedes albopictus* and *Aedes aegypti* pools [17]. The mosquito infection rate was supposedly very high since the authors reported 13 positive pools over 13 tested. We had the opportunity to test a few individual mosquitoes (7 *Aedes aegypti* and 4 *Aedes albopictus*) collected during the course of the epidemic around health care facilities in Brazzaville by the LNSP, and identified CHIKV in two *Aedes albopictus* specimens collected in Makélékélé (South Area).

The geographical distribution of CHIKV seroprevalence in March 2011 reveals the highest values in the North and South areas of Brazzaville city. These estimates were obtained from a population of blood donors and therefore are associated with usual limitations associated with this specific population, including an unbalanced gender ratio and the absence of individuals under the age of 18 years old. Fig. 2 shows that there is no clear relationship between the CHIKV seroprevalence status before the outbreak and the geographical distribution of incident cases in 2011.

The Central Area was still poorly impacted by the outbreak, possibly reflecting lower exposure to the bite of *Aedes* mosquitoes in the Central districts. However, amongst the two areas with high seroprevalence rates, one (North) was associated with a few incident cases whilst the other (South) hosted the vast majority of cases. This difference could obviously not be explained by different herd immunity levels (37.4% *vs* 40.0%) [33]. Regarding the spatial distribution of cases, our results are in accordance with WHO findings (72.3% of the total number of cases (7,279/10,066) were reported in the Makélékélé district).

Since the first CHIK descriptions made in Tanzania and Uganda in the 1960's, few studies have investigated CHIK outbreaks in Africa [3], [18], [19]. Several recent studies have suggested that CHIK clinical presentation may differ depending on the geographic location of the outbreak (*e.g.*, prevalence of persistent arthropathy or risk factors for severe disease [34], [35]. This may reflect different genetic backgrounds, co-morbidity factors or prior cross immunity [36], [37].

Here we report the clinical characteristics of laboratory confirmed Congolese CHIKV patients using a standardised questionnaire that allowed comparison with previous clinical studies, *e.g.* in Reunion Island. The incidence of the main clinical symptoms such as arthralgia, myalgia, dermatological signs and digestive disorder was within the range of previous reports [18].

Regarding arthralgia, we confirmed that the small joints of the upper member (wrist and hands) were more frequently affected and had the highest specificity to differentiate CHIK from other aetiologies. By contrast, the observation that CHIKV+ve patients had significantly more pruritus and burn under the feet than CHIKV-ve patients is not part of the canonical description of CHIK.

Differentiating CHIK from other febrile, treatable, aetiologies such as malaria or bacterial diseases is an important challenge. CHIK also shares some clinical signs with dengue and can be misdiagnosed in areas where dengue is common. Recently chikungunya diagnostic scores have been proposed from a cohort of outpatients during the Reunion Island outbreak [20]. The best results were obtained when clinical signs were associated with the lymphocyte count. Here, in the absence of blood cell count, we tested the clinical version of this score in Congolese suspected cases included within less than two days after the onset of the disease. Sensitivity (79% *vs* 76%) and specificity (72% *vs* 73%) values were similar to those obtained in the referent study [20]. These two studies were conducted in very different environments (*e.g.*, the socio-economic level was much higher, and the impact of other tropical diseases such as malaria was much lower in Reunion Island), but had in common the absence of dengue co-circulation. In contrast with the recent CHIK outbreak that occurred in Gabon [32] and in accordance with previous reports [17], we did not identify any dengue case amongst the 317 suspected cases investigated. In this epidemiological background, we found that the clinical score (Se, Sp, PPV, NPV: 78.6%, 72.4%, 45.0%, 92.2% respectively) permitted a more accurate follow-up of the evolution of the outbreak than the clinical criterion "fever and arthralgia" (Se, Sp, PPV, NPV: 85.7%, 44.9%, 30.8%, 91.7% respectively). The positive predictive value (45%) associated with possible/probable cases as defined by the clinical score is however low. This reflects the lower prevalence observed in the current cohort (22 *vs* 71% in the referent study) and indicates that this purely clinical score, even if it performs slightly better than the criterion "fever + arthralgia", would have been poorly contributory in medical practice to differentiate CHIKV+ve patients from others during the course of the Congolese outbreak.

This study provides confirmation that the CHIKV strains that currently circulate in Central Africa belong to the ECSA2 group. This group includes a number of strains collected in Central Africa before 1990 (and, anecdotally, the 2000 MH4 Indian strain which is highly similar to a 1982 Uganda strain and may represent a laboratory contamination, as previously suggested [28]) and Cameroonese and Gabonese strains identified in 2006–2007. Our phylogentic analysis includes full-length sequences of a 2007 Gabonese isolate and of a 2011 Congolese strain. They are closely related (Fig. 4) and both of them include adaptive mutations to *Aedes albopictus* in the envelope region. Strikingly, analysis of ECSA strains illustrates the propensity of CHIKV for dispersal and emergence in remote ecological environments. For example, numerous putative instances of CHIKV introduction in Singapore or Sri Lanka can be identified (Fig. 5). In addition, this phylogeny supports the hypothesis that, after 2006, the primary introduction and spread of CHIKV from Kenya in the Indian subcontinent and South-East Asia was linked to *Aedes aegypti*-associated strains (harbouring the E1 residue A226) [8]. In different locations, the selective pressure exerted on CHIKV through the constraint of having to replicate in a new vector (*Aedes albopictus*), was associated with independent events of mutation (A226V) constituting new examples of evolutionary convergence. For example, the tree is suggestive of at least two introductions of *Aedes aegypti*-associated strains in Sri Lanka, followed by the emergence of *Aedes albopictus*-adapted strains.

This phylogeny is helpful for proposing a credible evolutionary scenario for ECSA2 Central African isolates. It formally indicates that these viruses do not originate from ECSA viruses that have been circulating in the Indian Ocean Islands, the Indian subcontinent and Asia since 2005, which may have been brought in by viraemic travellers. Rather, they represent local descendants of viruses that have been circulating in Central Africa for decades and a scenario of convergent evolution that led to adaptation to *Aedes albopictus* can be proposed, which suggests that the colonisation of new territories by this mosquito is recent and massive.

This study suggests sustained circulation of CHIKV belonging to a specific lineage of the East/Central/South/African (ECSA) genotype in Central Africa. During the 2011 outbreak, the “arthralgia+fever” criterion had a good sensitivity for detection of CHIK cases, but low specificity and PPV values. A clinical score based on arthralgia (in hands and wrists) and myalgia was of poor medical interest.

The epidemiological and environmental determinants of CHIKV outbreaks in the region remain poorly characterised.
